# Use of Shared Decision-Making in Non–Muscle-Invasive Bladder Cancer: Protocol for a Scoping Review

**DOI:** 10.2196/90101

**Published:** 2026-04-30

**Authors:** Tullika Garg, Tierney Lyons, Kirstin Rabinowitz, Jazmine Gabriel

**Affiliations:** 1 Geisinger Health System, 100 N. Academy Avenue, Danville, PA, 17822, United States, 1 5702716328; 2 Geisinger Commonwealth School of Medicine, Scranton, PA, United States

**Keywords:** bladder neoplasms, patient-centered care, urology, decision-making, patient-centered communication

## Abstract

**Background:**

With a growing range of treatment options for non–muscle-invasive bladder cancer (NMIBC), the emergence of longitudinal patient-reported quality-of-life data, and an aging population managing the treatment burden of coexisting conditions, there is an increasing need for shared decision-making processes and tools to support urologists and patients in navigating complex treatment decisions. What remains unknown is how shared decision-making is currently incorporated into NMIBC treatment decisions and which factors inform key decisions in NMIBC.

**Objective:**

The objective of this scoping review is to map and characterize the existing literature on shared decision-making in NMIBC, with a focus on decision-making tools, decision points in the NMIBC cancer journey, and factors influencing patient and clinician decision-making.

**Methods:**

This scoping review protocol was designed in accordance with the Joanna Briggs Institute scoping review methodology. The final results will be reported in a subsequent publication in accordance with the PRISMA-ScR (Preferred Reporting Items for Systematic Reviews and Meta-Analyses Extension for Scoping Reviews) 2018 guidelines. A comprehensive search strategy will identify published, unpublished, and gray literature from databases (PubMed, Embase, CINAHL, PsycInfo, and Web of Science), professional society websites, and foundation websites. Eligibility criteria were determined based on the population, concept, and context framework. The population will include adults aged ≥18 years at the time of NMIBC diagnosis, with urothelial histology. The concept focuses on existing shared decision-making tools and patient- and urologist-level factors contributing to decisions. The context includes urology clinics in academic and community settings.

**Results:**

The literature search was conducted in September 2025. Title and abstract screening, full-text review, qualitative data coding, and quantitative data analysis are anticipated to be completed by October 2026.

**Conclusions:**

The treatment landscape for NMIBC is rapidly evolving, with a growing range of options, each with its own risks and benefits. This scoping review will help elucidate how shared decision-making is currently used in NMIBC treatment decisions and identify the key factors that are important to both urologists and patients. These data will guide future intervention development and research to enhance the quality of shared decision-making in NMIBC.

## Introduction

Bladder cancer is the sixth most common cancer in the United States, the fourth most common cancer in men, and disproportionately affects older adults (median age at diagnosis 73 years) [1]. Approximately 75% of diagnoses are non–muscle-invasive bladder cancer (NMIBC), which has a high recurrence rate (30%-70%) but a low risk of death [2]. Due to the high recurrence rate, NMIBC management involves multiple complex decision points over a years-long disease course, particularly as patients age and their health status changes due to frailty and other geriatric conditions such as urinary incontinence and cognitive impairment. Decisions vary across the cancer continuum (diagnosis, treatment, and survivorship) and can include choices regarding surveillance intensity, transurethral resection under general anesthesia, multiple intravesical therapies, and radical cystectomy.

Decisions regarding bacillus Calmette-Guérin (BCG)–unresponsive disease are particularly challenging, as patients must choose among alternative intravesical therapies (eg, gemcitabine, docetaxel, and nadofaragene firadenovec), systemic immunotherapy, and radical cystectomy (a major extirpative surgery to remove the bladder). Current guidelines from the American Urological Association mention using shared decision-making for BCG-unresponsive disease; however, there is no additional guidance on how shared decision-making should be conducted [2,3]. Furthermore, for patients with low- or intermediate-risk NMIBC, new intravesical chemoablative therapies and a new long-acting intravesical delivery system were approved by the US Food and Drug Administration in 2025, increasing the complexity of decision-making for other NMIBC disease states [4,5].

As the range of newly approved intravesical and systemic treatment options expands, alongside emerging longitudinal patient-reported data suggesting that patients with NMIBC who undergo radical cystectomy may experience better quality of life, and with an aging NMIBC population managing the treatment burden of coexisting conditions, there is a growing need for shared decision-making processes and tools to help urologists and patients navigate these increasingly complex decisions [6-10]. However, what remains unknown is how shared decision-making is currently incorporated into NMIBC treatment decisions and what factors inform these decisions. The overarching objective of this scoping review is to assess the extent of the literature on shared decision-making in NMIBC. A scoping review is appropriate for our research objective because we hypothesize that there are few shared decision-making tools for NMIBC treatment decisions; thus, delineating existing gaps will enable researchers to address this need. Specifically, we will describe (1) existing tools (if any) used for shared decision-making in NMIBC, (2) key decision points in NMIBC, and (3) factors that influence decisions from both patient and urologist perspectives.

## Methods

### Overview

This scoping review protocol was designed in accordance with the Joanna Briggs Institute (JBI) methodology for scoping reviews. The final results will be reported following the PRISMA-ScR (Preferred Reporting Items for Systematic Reviews and Meta-Analyses Extension for Scoping Reviews) 2018 checklist [11-13]. The scoping review protocol was developed a priori and has been registered on the Open Science Framework (5HTW8) [14].

### Ethical Considerations

Patients and the public are not involved in the design of this protocol. This scoping review protocol was reviewed by the Geisinger Institutional Review Board (GIRB 2025-0869), which determined that this project did not meet the definition of research as defined in 45 CFR 46.102(d).

### Definitions of Terms

We will use the National Quality Forum’s definition of shared decision-making: “a process of communication in which clinicians and patients work together to make optimal healthcare decisions that align with what matters most to patients” [15]. We will define NMIBC as urothelial carcinoma of the bladder that has not invaded the muscle layer of the bladder wall. It will be defined as American Joint Committee on Cancer (AJCC) stages 0a, 0is, or I or as TNM stages Ta, Tis, or T1; Nx or N0; and Mx or M0 [16]. We will also include variant histology that accompany urothelial carcinoma, such as squamous differentiation, sarcomatoid, plasmacytoid, glandular, micropapillary, and small cell or neuroendocrine differentiation.

A preliminary search of MEDLINE, the Cochrane Database of Systematic Reviews, and *JBI Evidence Synthesis* was conducted, and no current or ongoing systematic or scoping reviews on the topic were identified.

### Scoping Review Questions

We formulated the following research questions using the population, concept, and context (PCC) framework from the *JBI Manual for Evidence Synthesis*:

- What are the existing shared decision-making tools and/or decision aids available for NMIBC treatment decisions?
- Of the existing shared decision-making tools in NMIBC, what treatment decisions are they used to facilitate?
- What factors are most important to patients (eg, community, inpatients, and outpatients) and urologists when making NMIBC treatment decisions?

### Eligibility Criteria Based on the PCC Framework

#### Population

We will include adults aged ≥18 years at the time of NMIBC diagnosis. The focus of this scoping review will be on urothelial carcinoma histology primarily, but we will include additional variant histologies (squamous differentiation, sarcomatoid, plasmacytoid, glandular, micropapillary, and small cell or neuroendocrine differentiation). We will exclude children and young adults diagnosed with NMIBC (aged <18 years at diagnosis), nonurothelial histologies (pure squamous cell carcinoma, pure adenocarcinoma, and pure small cell or neuroendocrine carcinoma), and upper tract urothelial carcinoma. We will exclude literature that only describes participants with muscle-invasive, locally advanced, or metastatic bladder cancer, defined as AJCC stage ≥II or TNM stage T2-T4, or N1 or higher, or M1 or higher.

#### Concept

We will include any literature that describes existing shared decision-making tools and/or decision aids designed for NMIBC. We will also include any literature that addresses the concept of decision-making in NMIBC, shared decision-making in NMIBC, critical points for decision-making, or factors that urologists or patients consider important when making NMIBC treatment decisions. We will consider a variety of treatments such as surveillance cystoscopy, intravesical therapy, transurethral resection of bladder tumor, immunotherapy, and radical cystectomy. We will exclude literature that focuses exclusively on muscle-invasive bladder cancer. The treatment options and management decisions for muscle-invasive bladder cancer are very different from those for NMIBC and are beyond the scope of this review.

#### Context

We will include worldwide literature from urology clinic settings, as well as academic and community practices and hospitals.

### Types of Sources

This scoping review will consider both experimental and quasi-experimental study designs, including randomized controlled trials, nonrandomized controlled trials, before-and-after studies, and interrupted time-series studies. In addition, analytical observational studies, including prospective and retrospective cohort studies, case-control studies, and analytical cross-sectional studies, will be considered for inclusion. This review will also consider descriptive observational study designs such as case series, individual case reports, and descriptive cross-sectional studies for inclusion. Qualitative studies will also be considered, including, but not limited to, designs such as phenomenology, grounded theory, ethnography, qualitative description, action research, and feminist research. In addition, systematic reviews that meet the inclusion criteria will be considered. We will exclude protocols without outcomes, commentaries, and editorials.

### Search Strategy

The search strategy was designed and executed in collaboration with a medical librarian (TL). The PubMed search strategy is provided in Textbox 1. The search strategy will aim to locate both published and unpublished studies from 2010 to September 2025. First, an initial limited search of MEDLINE (PubMed) and CINAHL (EBSCO) was undertaken to identify articles on the topic. The text words contained in the titles and abstracts of relevant articles, and the index terms used to describe the articles, were used to develop a full search strategy. The search strategy, including all identified keywords and index terms, will be adapted for each included database and/or information source. The reference list of all included sources of evidence will be screened using the title and abstract screening flowchart for additional studies.

Textbox 1. **PubMed search strategy (from January 1, 2010, to September 2025).**

- Date searched: September 30, 2025
- Search query: (“decision making, shared”[MeSH] OR “decision making”[MeSH] OR decision making[tiab] OR “decision support techniques”[MeSH] OR decision support[tiab] OR decision aid*[tiab] OR patient decision tool*[tiab] OR “patient preference”[MeSH Terms] OR Patient Preference*[tiab] OR patient experience[tiab]) AND (“non-muscle invasive bladder neoplasms”[MeSH Terms] OR non muscle invasive bladder cancer[tiab] OR non muscle invasive bladder neoplasm*[tiab] OR Nonmuscle-invasive Bladder Cancer[tiab] OR Nonmuscle-invasive Bladder neoplasm*[tiab] OR “mycobacterium bovis”[MeSH Terms] OR mycobacterium bovis[tiab] OR ((bacillus calmette guerin[tiab] OR BCG[tiab]) AND (failure OR unresponsive OR refractory OR intolerant))) AND (“therapeutics”[MeSH Terms] OR therapeutics[tiab] OR treatment*[tiab] OR “therapy”[Subheading] OR therap*[tiab] OR “cystectomy”[MeSH Terms] OR radical cystectomy[tiab] OR Intravesical therapy[tiab] OR “immunotherapy”[MeSH Terms] OR immunotherap*[tiab] OR systemic therap*[tiab] OR “cystoscopy”[MeSH] OR cystoscop*[tiab] OR “transurethral resection of bladder”[MeSH] OR transurethral resection of bladder[tiab] OR “Immune Checkpoint Inhibitors”[Mesh] OR Immune Checkpoint Inhibit*[tiab] OR Immune Checkpoint Blocker*[tiab] OR Cytotoxic T Lymphocyte Associated Protein 4 Inhibitor*[tiab] OR CTLA-4 Inhibitor*[tiab] OR Programmed Cell Death Protein 1 Inhibitor*[tiab] OR PD 1 Inhibitor*[tiab] OR Immune Checkpoint Blockade*[tiab] PD L1 Inhibitor*[tiab] OR Programmed Death-Ligand 1 Inhibitor*[tiab] OR PD 1 PD L1 Blockade*[tiab] OR Immune Checkpoints Inhibit*[tiab])
- Records retrieved: 173

Only studies published in English from 2010 to the present will be included. The most recent American Urological Association guideline for NMIBC management underwent a major update in 2016 that defined a risk stratification algorithm, risk-stratified surveillance protocols, and guidance on shared decision-making and new treatments. We opted to look back to 2010 to capture conversations in the literature that may have prompted the guideline update.

The databases to be searched include PubMed, Embase, CINAHL, PsycInfo, and Web of Science. Sources of unpublished studies and gray literature to be searched include conference abstracts and related organizations (eg, Bladder Cancer Advocacy Network, American Urological Association, Fight Bladder Cancer UK, Urology Cares Foundation, International Bladder Cancer Group, American Cancer Society, International Society of Geriatric Oncology, European Association of Urology, Action Bladder Cancer, Bladder Cancer Awareness Australia, International Bladder Cancer Network, World Bladder Cancer Patient Coalition, and BEAT Bladder Cancer Australia).

### Source of Evidence Selection

**Figure 1.**
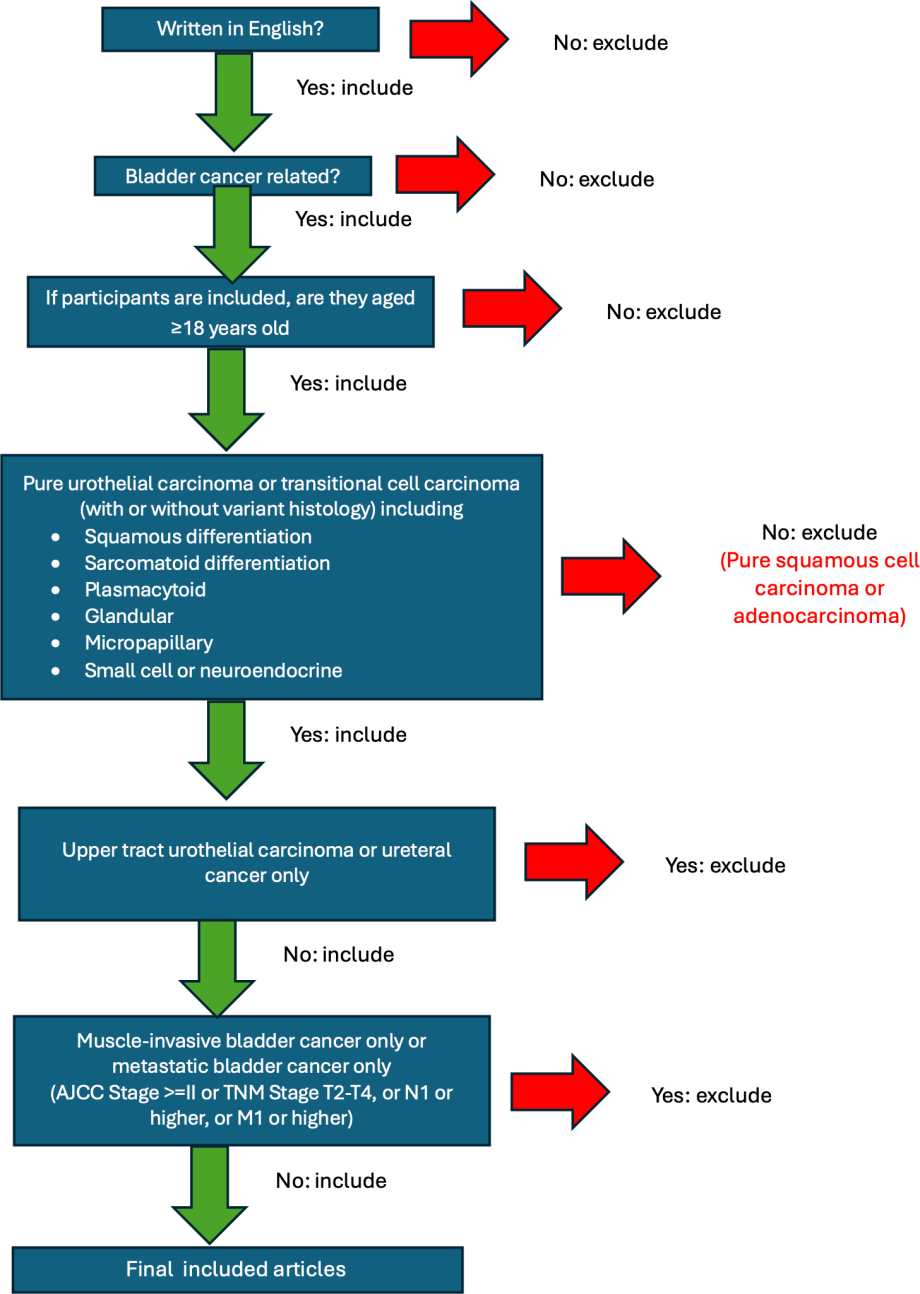
Flowchart for title and abstract screening. AJCC: American Joint Committee on Cancer.

Following the search, all identified citations will be collated and uploaded into Covidence (Veritas Health Innovation), and duplicate records will be removed. We developed a flowchart to aid reviewers in title and abstract screening (Figure 1). Following a pilot test of 10 records (interrater agreement 75%), titles and abstracts will then be screened by 2 independent reviewers for assessment against the inclusion criteria of the review. Potentially relevant sources will be retrieved in full, and their citation details will be imported into Covidence and screened by 2 independent reviewers. The full text of selected citations will be assessed in detail against the inclusion criteria by 2 or more independent reviewers. Reasons for exclusion of sources of evidence at the full-text stage that do not meet the inclusion criteria will be recorded and reported in the scoping review. Any disagreements that arise between the reviewers at each stage of the selection process will be resolved through discussion to reach a consensus, and if necessary, a third reviewer will act as a tiebreaker. Consistent with scoping review methodology and goals, we will not assess the methodological quality of the included studies. The results of the search and the study inclusion process will be reported in full in the final scoping review manuscript and presented in a PRISMA (Preferred Reporting Items for Systematic Reviews and Meta-Analyses) flow diagram [12].

### Data Extraction

Data will be extracted from papers included in the scoping review by 2 or more independent reviewers using a data extraction tool developed by the reviewers. Each study or gray literature document will be reviewed by 2 reviewers to ensure consistency in data extraction. The data extracted will include specific details about the participants, concept, context, study methods, and key findings relevant to the review questions. We will pilot the data extraction tool on 3 papers. If there are inconsistencies in data extraction during the pilot, we will modify the tool as needed. We will report modifications to the tool in the final scoping review manuscript. A draft extraction form is provided in Multimedia Appendix 1. Any disagreements that arise between the reviewers will be resolved through discussion or with input from an additional reviewer. We will not contact study authors to request missing or additional data.

### Data Analysis and Presentation

To meet the objectives of this scoping review and to answer our research questions, we will analyze both quantitative and qualitative data. We will calculate descriptive statistics and summarize quantitative data from existing shared decision-making tools and key decision points in NMIBC management in a table and a figure, respectively. We will summarize data from qualitative studies to identify factors important to urologists and patients when making NMIBC treatment decisions.

**Figure 2.**
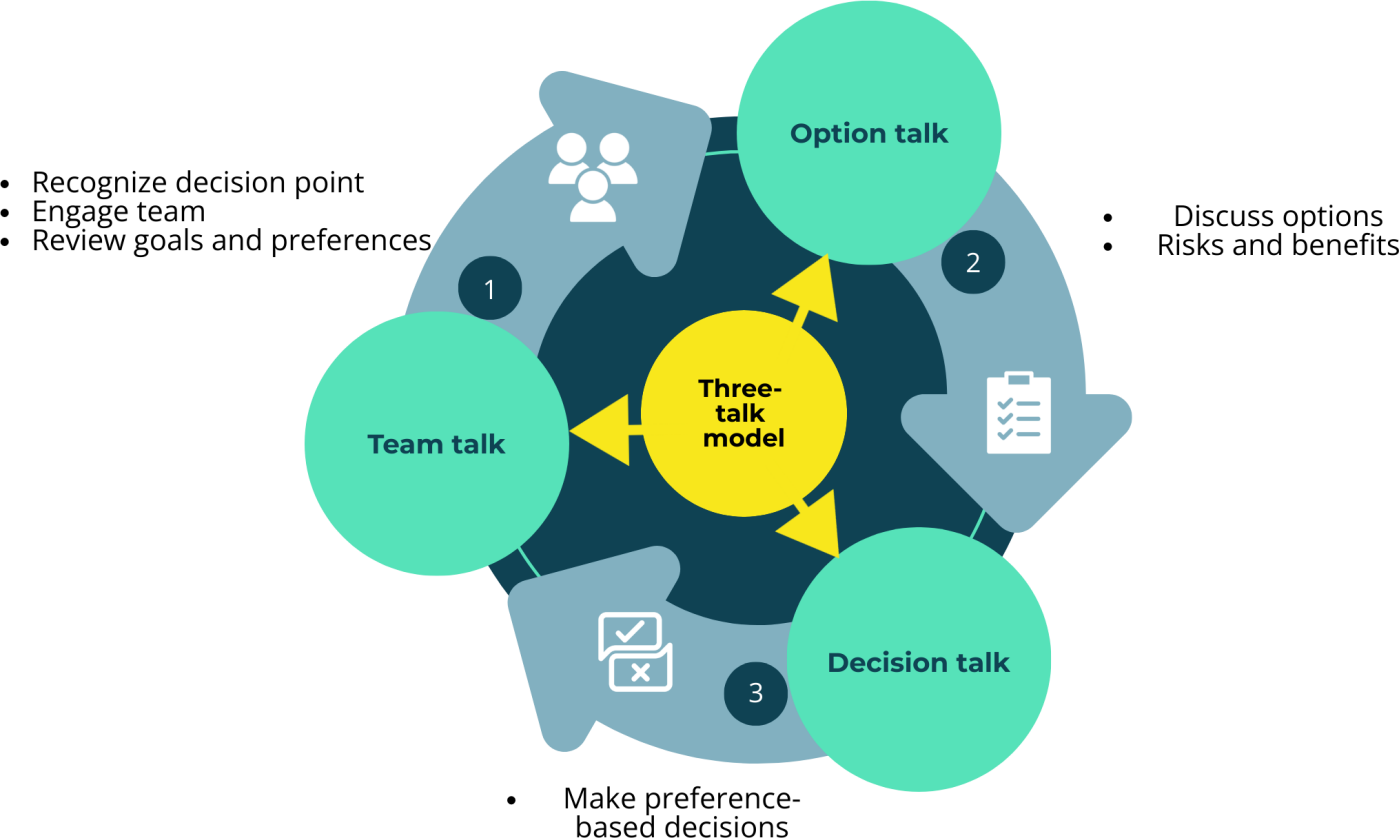
Elwyn three-talk model of shared decision-making.

We will analyze the qualitative data using directed content analysis, using a codebook based on the 2017 update of the Elwyn three-talk model of shared decision-making (Figure 2) and the categories defined in the scoping review PCC framework [17,18]. The three-talk model is a simple, 3-step shared decision-making approach vetted by clinicians, including urologists. Inductive codes will be added as needed to capture emerging concepts not covered by the deductive framework described above. We will use ATLAS.ti (Lumivero) to organize, code, and analyze the data.

Two coders will independently code 20% of the data to establish the preliminary codebook. The codebook will be refined iteratively through review and discussion between the 2 coders. The 2 coders will apply the revised codebook to another 20% of the data, and we will assess interrater reliability. Disagreements will be discussed, and the codebook will be further refined. The process will continue until the coders reach acceptable interrater reliability (κ>0.7) [19]. The remaining data will be coded twice (once by each coder) to ensure stable coding across all included studies. The full scoping review team will review codes and coding patterns to confirm stable coding across the data and will then collate codes to describe the themes that emerge.

The qualitative data will be summarized in narrative form and in a table with representative quotes for identified factors, which may be grouped further into broader themes, categories, or relationships in a figure.

## Results

**Figure 3.**
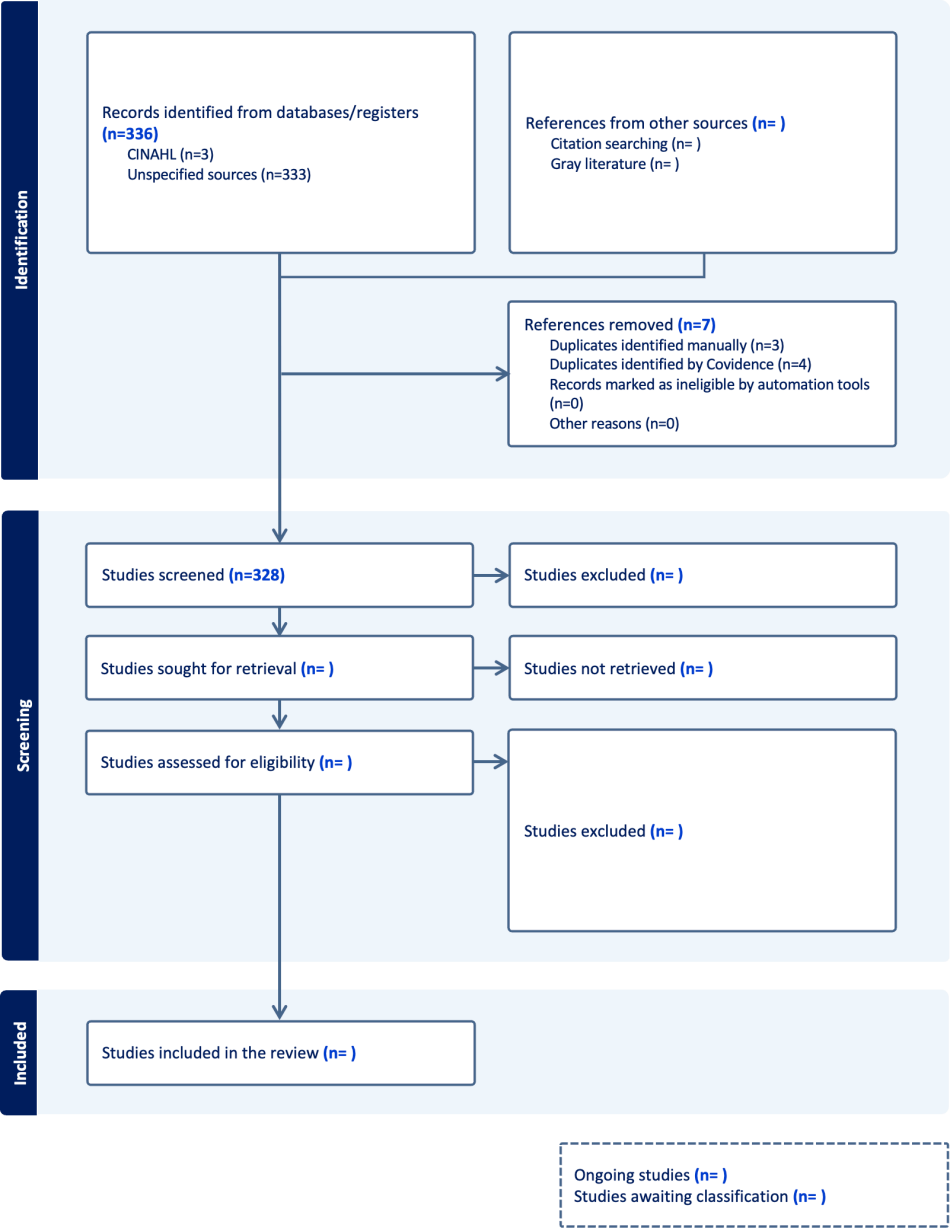
PRISMA (Preferred Reporting Items for Systematic Reviews and Meta-Analyses) flowchart.

The literature search was conducted in September 2025, and we identified 329 publications after removing duplicates in Covidence. Title and abstract screening, full-text review, qualitative data coding, and quantitative data analysis are anticipated to be completed by October 2026 (Figure 3).

## Discussion

### Anticipated Findings

We anticipate that the findings from this scoping review will inform future interventions, such as shared decision-making tools, that rigorously incorporate structured shared decision-making conversations into NMIBC management. Much of the prior research on decision aids and shared decision-making in bladder cancer has focused on muscle-invasive disease (stage II or higher) and major decisions regarding chemotherapy/immunotherapy, radical cystectomy, and various urinary diversion options. In contrast to the single major decision usually required for muscle-invasive disease, NMIBC requires more chronic decision-making due to frequent recurrences that impact patients over time. As the treatment landscape for NMIBC continues to evolve, with multiple new drugs (both systemic medications and intravesical therapies) that have unique side effect profiles and treatment burdens, urologists will need to be better equipped to navigate patients and families through these options using shared decision-making. This scoping review will define pivotal decision points in the NMIBC care journey and characterize the factors that urologists and patients consider when weighing different treatment options. The data from this scoping review will help us design interventions and target them to the NMIBC treatment decisions where shared decision-making is needed most. We suspect that there is limited data on shared decision-making in NMIBC; therefore, we also anticipate identifying significant knowledge gaps that still need to be filled.

### Limitations

Our protocol must be considered within the context of certain limitations. Due to time and resource constraints, we will not be able to include manuscripts written in other languages, which could bias the generalizability of our results. Similarly, we will not contact authors for additional data or clarifications, which could lead to relevant data being missed. Many of the new treatments have been approved in only the past 1 to 2 years, and there may not be enough long-term and patient-reported data on these new options. We will follow the established scoping review methodology as rigorously as possible to mitigate these limitations.

### Conclusions

This scoping review will evaluate the current landscape of shared decision-making in NMIBC. We will compile existing tools and delineate the factors and decision points most important to patients and urologists when making NMIBC treatment decisions. We anticipate that the results of this scoping review will guide intervention development and future research to fill knowledge gaps.

## Supplementary material

10.2196/90101 Multimedia Appendix 1 Draft data extraction instrument.

## Abbreviations

AJCC: American Joint Committee on Cancer
BCG: bacillus Calmette-Guerin
JBI: Joanna Briggs Institute
NMIBC: non–muscle-invasive bladder cancer
PCC: population, concept, and context
PRISMA: Preferred Reporting Items for Systematic Reviews and Meta-Analyses
PRISMA-ScR: Preferred Reporting Items for Systematic Reviews and Meta-Analyses Extension for Scoping Reviews
