# Supplementary material for: Use of Shared Decision-Making in Non–Muscle-Invasive Bladder Cancer: Protocol for a Scoping Review
Source: JMIR Res Protoc. 2026 Apr 30;15:e90101. doi: 10.2196/90101 (PMC13131830; doi:10.2196/90101)
Supplement: Multimedia Appendix 1 [file resprot-v15-e90101-s001.docx]

**Multimedia Appendix: Draft Data Extraction Instrument**

| **Article Details** | |
| --- | --- |
| Study Title |  |
| Author |  |
| Year of Publication |  |
| Country |  |
| Publication Type (Manuscript, Conference Abstract, Grey Literature) |  |
| Study type |  |
| Objective |  |
| **Participants** | |
| Age |  |
| Who was recruited (patients, urologists, both) |  |
| Sample size |  |
| NMIBC AJCC Stage |  |
| NMIBC Histology |  |
| AUA Risk Category (low, intermediate, high, very high, unknown) |  |
| Addresses BCG Unresponsive Disease |  |
| **Concept** | |
| Shared decision-making tool |  |
| Decision aid tool |  |
| Shared Decision-Making Tool/Decision Aid was validated (yes/no) |  |
| Decision point addressed |  |
| Factors considered by patients when making decisions |  |
| Factors considered by urologists when making decisions |  |
| **Context** | |
| Urology clinic |  |
| Hospital |  |
| Community |  |
| Academic |  |
| **Other** | |
| Author-identified limitations |  |
| Author-identified research opportunities |  |
